# X-ray Image-Based Pose Estimation of a Joint-Encoded Spinal Surgical Positioning Arm

**DOI:** 10.7759/cureus.87212

**Published:** 2025-07-03

**Authors:** Yi-Hsin Tsai, Chih-Min Yang, Xiu-Yun Xiao, Hsuan Han Fang, Yi-Chi Pan, Hao-Kai Chou, Shih-Hao Huang, Ming-Hong Chen

**Affiliations:** 1 Division of Neurosurgery, Department of Surgery, Far Eastern Memorial Hospital, New Taipei City, TWN; 2 Department of Research and Development, SAVFE Co. Ltd., New Taipei City, TWN

**Keywords:** accuracy, navigation system, positioning system, radiation exposure, spine surgery, surgical robotics

## Abstract

Minimally invasive spinal surgery requires accurate and efficient surgical tool positioning; however, current optical or navigation-assisted systems can be costly, complex, or expose patients to increased radiation. To address these challenges, we propose and evaluate a novel passive spinal surgical positioning arm with X-ray image-based pose estimation capability. The system estimates the 6-degree-of-freedom pose of the arm using radiographic landmarks extracted from a single intraoperative X-ray image.

In our experiment, a surgeon performed single-level pedicle screw placement on three sawbones spine models. The estimated positioning accuracy achieved a mean translational error of 1.08 ± 0.40 mm. The average setup time was 2.3 ± 0.9 minutes, and the surgical operation time was 4.2 ± 1.8 minutes. Only 6.75 ± 0.95 X-ray images were required per procedure, resulting in an average radiation dose of 0.27 ± 0.04 mGy.

These results demonstrate that our system can potentially reduce radiation exposure and surgical duration while maintaining high positioning accuracy. Further clinical validation is required to confirm its effectiveness in real-world surgical settings.

## Introduction

Minimally invasive spine surgery and pedicle screw placement have become widespread treatments for many spinal conditions with proven benefits [1]. However, precise instrument positioning and navigation remain significant challenges in spine surgery, affecting both clinical outcomes and operational efficiency.

The traditional fluoroscopy-guided pedicle screw placement, while a standard procedure, still faces numerous challenges. According to the literature, the deviation rate for traditional pedicle screw placement can reach 16.4%, with some cases requiring revision surgery [2]. Surgeons often depend on X-rays or CT scans to identify the precise location of instruments within the patient's body. However, this approach increases radiation exposure for both the surgical team and the patient and may lead to errors due to image quality or positioning inaccuracies [2,3]. Research shows that excessive X-ray exposure may bring long-term health risks, especially for medical personnel working with high radiation exposure [4]. Therefore, to address these issues, various surgical robot systems and navigation systems have been developed in the market.

Compared to traditional fluoroscopy-guided procedures, robot-assisted systems and navigation systems can improve surgical precision and reduce radiation dose exposure [5,6]. La Rocca et al.'s research indicated that the use of CT navigation systems (CTNav) significantly improved pedicle screw placement accuracy compared to traditional C-arm fluoroscopy-guided techniques. Screw placement accuracy was assessed postoperatively using a CT scan, employing the Gertzbein and Robbins classification (grades A-E) in this study. In the CTNav group, 96.4% of screws were classified as A or B grade, compared to 92% in the fluoroscopy-guided group. The CTNav group had no E-grade screws (completely misaligned), while the fluoroscopy-guided group had 16% [7]. Robot-assisted surgery also demonstrates significant accuracy advantages. According to Torii et al.'s research, using the Mazor X Stealth Edition robot (Mazor X Stealth Edition, Medtronic Inc., Dublin, Ireland) dramatically reduced screw deviation rates, with accuracy reaching 85-100% [8].

By utilizing preoperative CT scans for intraoperative positioning, CT navigation technology can eliminate the need for C-arm imaging and reduce radiation exposure for the surgical team [7,9]. Besides CT navigation, robot-assisted surgery can also effectively reduce the time spent on radiation exposure by the surgical team. In Torii et al.'s research, the robot group's total radioactive image capture time was only 29.6% of that of the traditional freehand group [8], significantly reducing the need for radioactive image capture. In Pennington et al.'s study, surgeon radiation exposure was lower when using navigation systems and robot-assisted systems compared to a fluoroscopy guide [10]. In Chang et al.'s study, a lower effective dose for patients is reported for navigation systems than fluoroscopy guides in long-segment surgery [11].

Although surgical robot systems and navigation systems have demonstrated significant advancements in surgical precision and a reduction in radiation dose exposure, several challenges remain unresolved. Research shows that even experienced surgeons may initially encounter difficulties when first using robotic systems, requiring additional training and preparation to become proficient [12]. In Torii et al.'s research, the robotic system's operation time might be slightly longer in the early stages and need accumulated experience to help reduce screw insertion time from approximately 5.5 minutes per screw to about four minutes [8].

Setup time is also a crucial indicator for surgical assistance systems. Ding et al.'s research demonstrated that using an O-arm navigation system, including image scanning and navigation setup, had an average setup time (from skin incision to reference frame installation) of 28.3 minutes [13]. Robotic systems may require longer setup times due to the integration of preoperative CT images and calibration of the navigation system [8].

Moreover, mainstream surgical robot systems and navigation systems primarily use optical navigation [14], which presents specific limitations in clinical practice. Optical tracking systems can be easily affected by environmental factors, such as changes in light or interference from blood-stained markers. Additionally, since positioning depends on optical tracking, instrument marker points must remain constantly visible to the tracker, requiring users to pay attention to instrument orientation or adjust their usage habits, which can consume considerable time for setup and calibration. These limitations not only affect workflow efficiency but may also impact surgical precision when the line of sight is interrupted.

Despite these advancements, a significant gap remains in developing a positioning system that can (1) function independently of optical tracking systems, (2) reduce intraoperative radiation exposure, (3) maintain high accuracy, and (4) minimize setup time and workflow disruption. Current systems often achieve some of these goals at the expense of others, creating a need for an integrated solution that addresses all these challenges simultaneously.

In response to these challenges, this research has developed a novel positioning system that combines a multi-link mechanism with encoders for spatial tracking. Specifically, this approach aims to (1) design a multi-link mechanism with encoders capable of accurately tracking surgical instrument positions in 3D space, (2) develop a real-time 2D X-ray navigation method that reduces the need for multiple fluoroscopic images, and (3) evaluate the system's accuracy, setup time, and workflow efficiency compared to conventional navigation systems.

This study encompasses laboratory validation using a spine phantom. The system's performance is assessed through quantitative measurements of positioning accuracy, setup time, and radiation exposure metrics.

In summary, while current surgical navigation technologies have significantly contributed to precision and surgical assistance, numerous challenges remain. This paper aims to explore the design, application, and effectiveness of our novel device in enhancing surgical efficiency and accuracy, compare it with existing equipment, and provide a new perspective for the future development of surgical navigation technology.

## Technical report

ArmSure fluoroscopic positioning system

**Figure 1:**
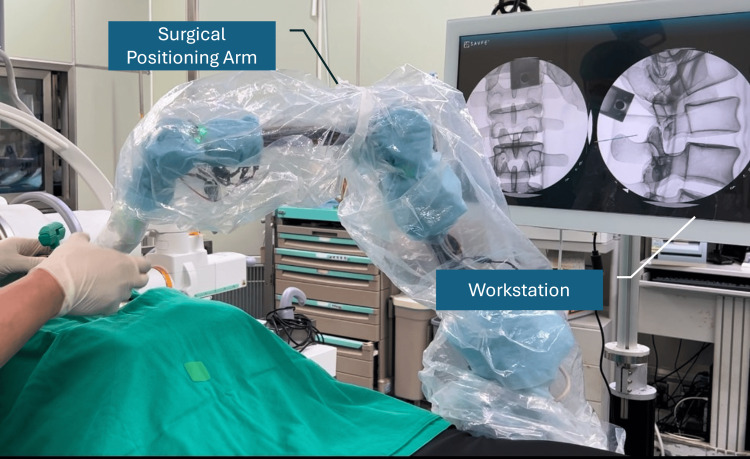
Overview of the ArmSure fluoroscopic positioning system, including the passive surgical positioning arm and workstation with integrated display and processing unit. The system is designed for X-ray-guided spinal navigation without optical tracking

The ArmSure fluoroscopic positioning system includes a workstation (containing a screen and computer host), a surgical positioning arm (a passive arm with seven degrees of freedom), a customized calibrator, and surgical instruments, such as a trocar (Argon Medical Device, Plano, USA), as shown in Figure 1.

The surgical positioning arm consists of seven axes, with an additional redundant degree of freedom compared to typical mechanical arms to provide a more seamless operation experience similar to a human arm. Each rotary axis is equipped with a brake system and an encoder. By recording the rotation of each axis through the encoders, the system can calculate the position of the arm's end effector, thereby achieving positioning functionality.

Previous studies have highlighted the advantages of multi-joint linkages combined with encoders in achieving high positioning accuracy [15,16]. Inspired by this design concept, we have developed a positioning system tailored for use in operating rooms. Since positioning is done through the arm and does not require an optical tracking system, there are no optical occlusion problems and no issues with marker contamination or positioning inaccuracies due to lighting conditions. Users only need to ensure they are gripping the instrument securely during positioning, without altering their normal usage habits. Additionally, this positioning system does not include motors or other active components and only provides arm unlocking or locking states through brakes. The design of the passive arm enables the reduction of the positioning system's size and weight.

This system requires only a small number of X-ray images, or even just a single X-ray image, to perform calibration and positioning effectively. The workstation will then display 2D X-ray images taken during surgery. By using the positioning arm to capture the instrument's position, it can project the instrument onto the X-ray image, simulating how the instrument would appear when taking an X-ray during the surgical procedure, thus navigating the surgical process.

**Figure 2:**
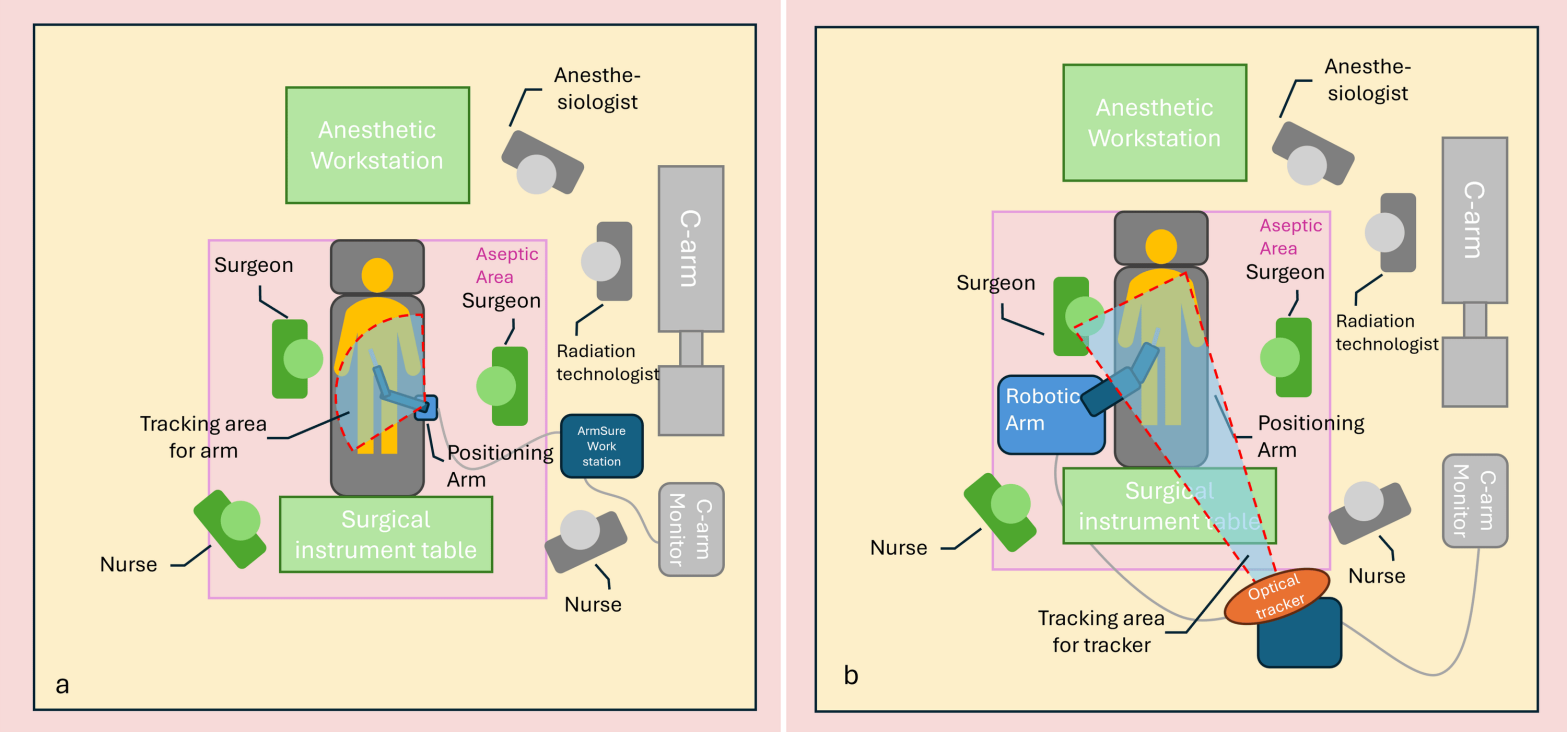
(a) Operation room setup for the ArmSure system, showing its compact footprint and minimal obstruction. (b) Comparative layout for a conventional robotic navigation system, which requires more space and a clear optical line-of-sight for tracking markers Image Credit: Hsuan-Han Fang

The entire design of the positioning system requires only a small workspace (Figure 2a), making it convenient to use in various surgical settings, including operating rooms in medical centers or ambulatory surgery centers. Compared to other optical navigation and robot-assisted surgical devices (Figure 2b), our positioning system is more compact and lighter in weight. It is more portable and is easier to set up. These comparisons will be elaborated upon in subsequent sections.

ArmSure installation and positioning workflow

**Figure 3:**
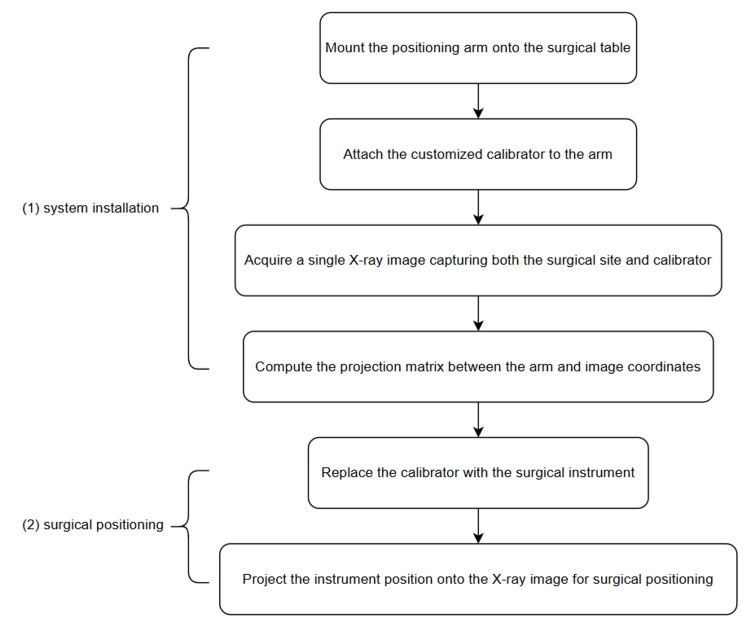
Experimental workflow for system setup and surgical navigation. The process is divided into two main stages: (1) system installation, where the positioning arm and customized calibrator are mounted and calibrated using a single X-ray image, and (2) surgical positioning, where the calibrator is replaced with the surgical instrument, and its real-time position is projected onto the X-ray image for surgical positioning

The overall process is divided into two main parts: system installation and surgical positioning (Figure 3).

**Figure 4:**
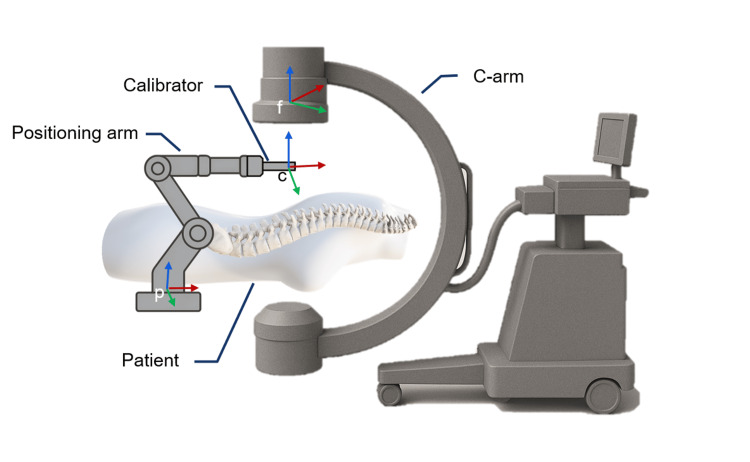
Coordinate system configuration for spatial transformation. The positioning arm (frame p), customized calibrator (frame c), and fluoroscopic image coordinate system (frame f) are shown. The projection matrix is computed by identifying corresponding landmarks between the calibrator and the 2D image, enabling transformation from the physical tool space to the fluoroscopic image space. The labeled coordinate frames (p, f, c) correspond to the notation used in the calibration and transformation process described in the installation and positioning workflow section 2D: two dimensional Image Credit: Authors; Microsoft Office (Microsoft Corp., Redmond, WA, USA) built-in 3D models were used to create the original illustrations.

During the system installation process, the positioning arm is mounted on the operation table, and its end is connected to the calibrator, which contains metal balls for calibration. During the calibration, we aimed to calculate the projection matrix $T_{p\to f}$ between the positioning arm and the 2D fluoroscopic image (Figure 4) using the formula:

$T_{p\to f}=T_{p\to c}\ast T_{c\to f}$

Therefore, we need to get the transformation matrix $T_{c\to f}$ between the calibrator and 2D fluoroscopic image and the transformation matrix $T_{p\to c}$ between the positioning arm and calibrator. $T_{p\to c}$ is known since the calibrator is connected to the positioning arm. To get $T_{c\to f}$ our system requires a single X-ray image that simultaneously captures both the surgical site and the calibrator. By analyzing the correspondence between feature points on the calibrator in both the image coordinate system and the arm coordinate system, we can compute $T_{c\to f}$. The spatial correspondence between features identified in the X-ray image coordinate system and their known positions in the surgical arm coordinate system was computed using the direct linear transformation technique. This approach enables precise mapping between the two coordinate spaces through the following mathematical formulation:

$\begin{bmatrix}u \\v \\1\end{bmatrix}=T_{p\to f}\begin{bmatrix}X \\Y \\Z \\1\end{bmatrix}=\begin{bmatrix}T_{11} &T_{12} &T_{13} &T_{14} \\T_{21} &T_{22} &T_{23} &T_{24} \\T_{31} &T_{32} &T_{33} &T_{34} \end{bmatrix}\begin{bmatrix}X \\Y \\Z \\1\end{bmatrix}$

where X, Y, and Z were the positioning data from the positioning arm, and u and v were the pixel data from C-arm 2D images.

In the surgical positioning phase, the calibrator at the arm's end is replaced with a surgical instrument. This enables the system to project the instrument's real-time position onto the previously captured X-ray image, thereby guiding the placement and movement of the surgical instrument.

Experimental procedure

**Figure 5:**
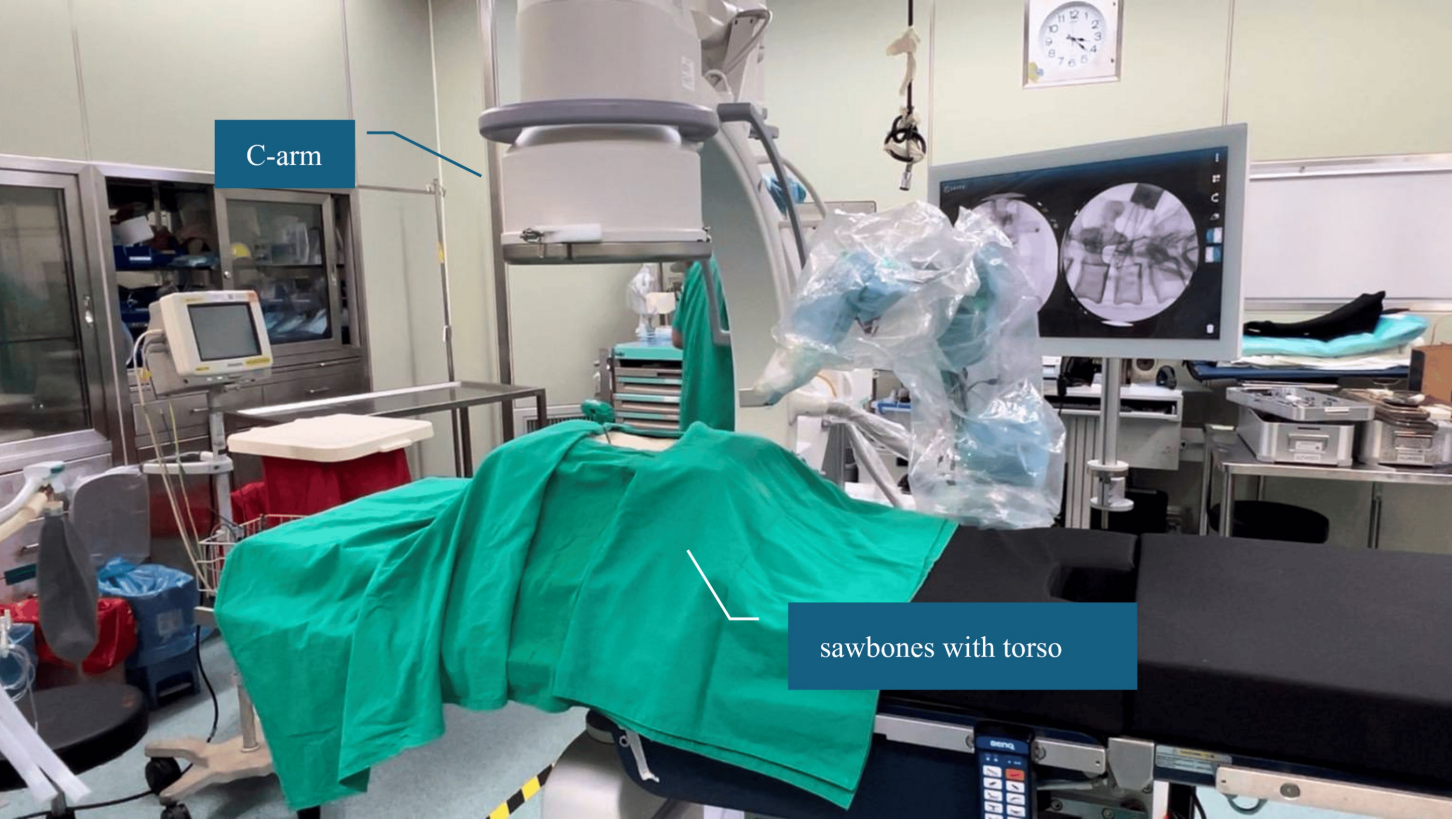
Experimental setup using a sawbones spine model (L1–L5) with torso, positioned on an operating table and imaged using a C-arm system to simulate intraoperative conditions

In this study, we evaluated and discussed three aspects: the surgical time required for positioning the arm, the number of intraoperative C-arm images acquired, and the accuracy of the positioning arm. For the experiments on surgical time and the number of intraoperative C-arm images acquired, we prepared three sets of sawbones spine models (Sawbones, Vashon, USA), with vertebral segments from L1 to L5 and torso, to simulate actual surgical scenarios (Figure 5) and an OEC One C-arm (GE HealthCare, Illinois, USA) to capture radiographic images. A surgeon performed a single vertebral pedicle screw insertion procedure, from equipment setup to surgery completion. During the process, we recorded two intervals of time: the setup time and the operation time. The setup time is defined as the duration from the preparation of the equipment to the start of the surgery. During this period, we will set up the positioning arm on the operating table, capture C-arm images, and perform calibration. The operation time is measured from the start of the surgery to the completion of the single vertebral pedicle screw insertion. Additionally, the total number of intraoperative C-arm images acquired during the procedure was recorded.

**Figure 6:**
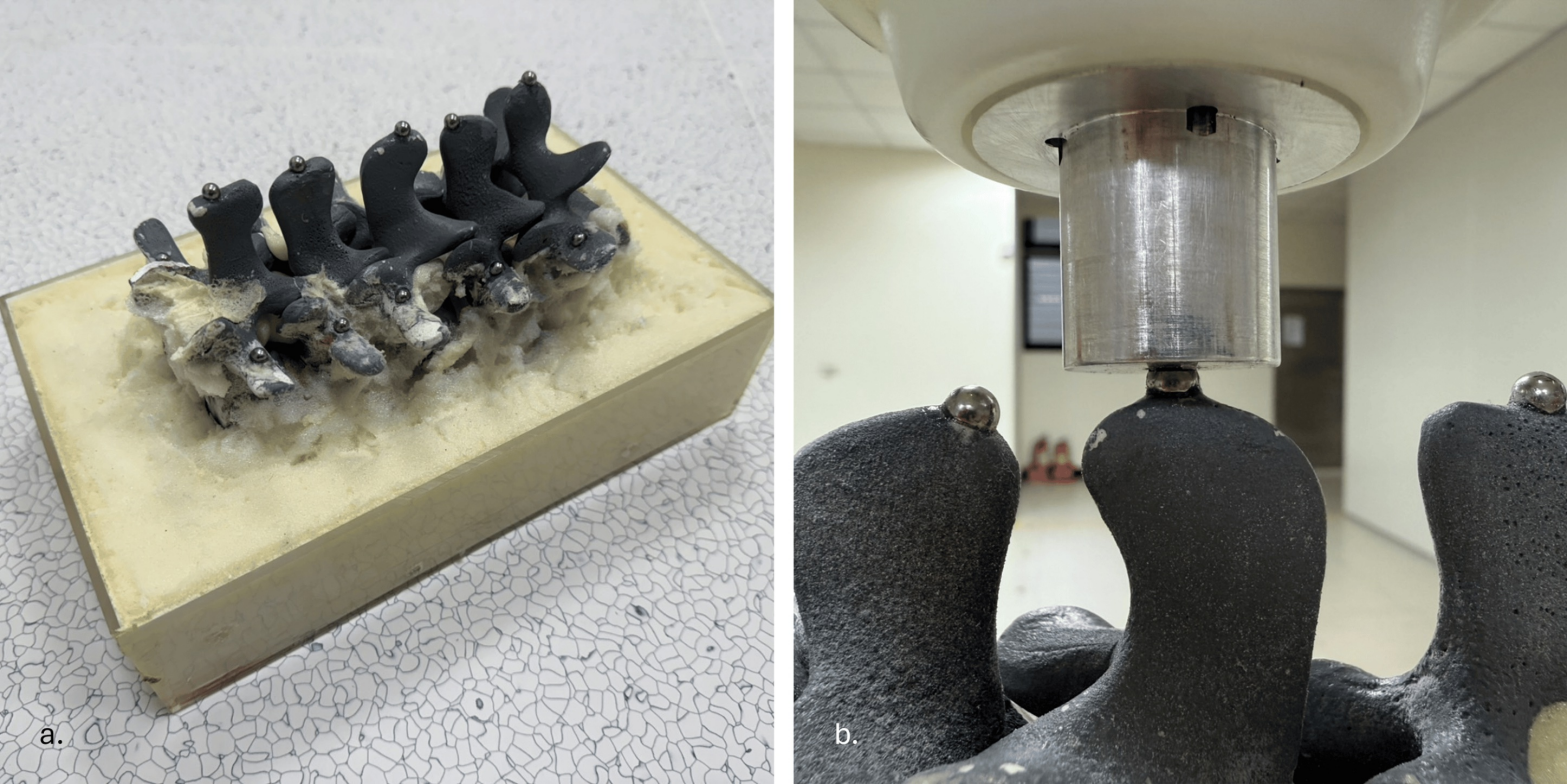
(a) Sawbones spine model with 15 steel ball markers fixed to anatomical landmarks. (b) Positioning arm equipped with a custom hemispherical probe used to measure each steel ball's 3D location for accuracy validation 3D: three dimensional

**Figure 7:**
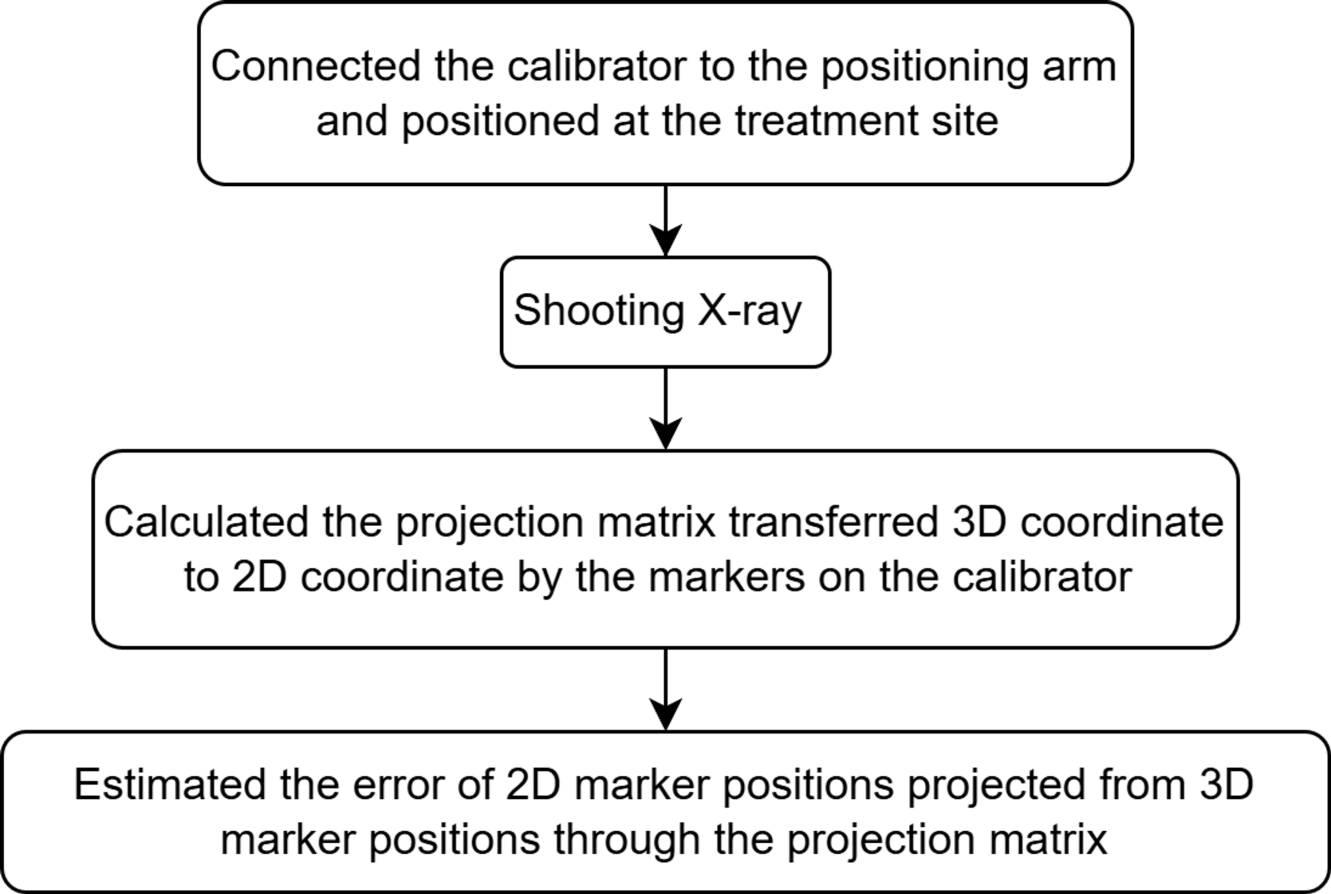
Procedure for validating image calibration accuracy by projecting known 3D points from the calibrator onto the X-ray image using the computed projection matrix and calculating 2D projection errors 3D: three dimensional, 2D: two dimensional

**Figure 8:**
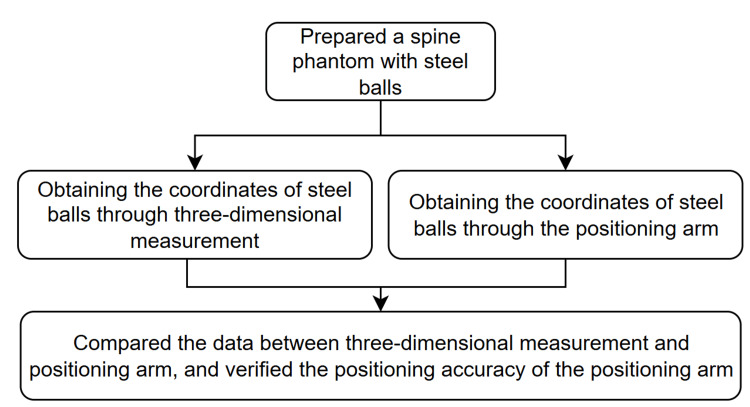
Workflow for verifying the positioning accuracy of the surgical arm using a phantom embedded with steel ball markers. The arm is equipped with a custom hemispherical measurement attachment to touch each marker. The measured 3D coordinates are compared with ground truth positions obtained from a CMM to quantify the arm’s intrinsic spatial accuracy 3D: three dimensional, CMM: coordinate measuring machine

To verify the positioning accuracy of the positioning arm, we designed an experimental protocol to provide quantitative data verifying the positioning arm's precision. The experiment utilizes a Sawbones spine model (Sawbones, Vashon, USA), featuring vertebral segments from L1 to L5. The phantom is positioned with the anterior side facing down and the posterior side facing up, fixed within an acrylic box using foam material. Steel balls with a diameter of 5 mm are fixed onto the sawbones spine model, with a total of 15 balls in place (Figure 6a). The fixation points were distributed as follows: five steel balls were attached to the spinal processes of each vertebral segment, five steel balls were fixed at the left pedicle screw entry points of each vertebral segment, and five steel balls were fixed at the right pedicle screw entry points of each vertebral segment. The entire experiment is divided into two parts for verification: (1) accuracy of image calibration and (2) accuracy of surgical positioning. By adding the measurement errors from these two parts, the overall system accuracy can be determined. The verification process is illustrated in Figure 7 and Figure 8.

Accuracy of image calibration

In the method proposed in this paper, users determine the actual spatial position of instruments by projecting real-time instrument images onto a 2D perspective X-ray on the screen. Therefore, the error in the transformation between 2D X-ray coordinates and 3D spatial coordinates represents the discrepancy between the actual instrument position and the positioning information displayed in the image. Using the customized calibrator, images containing the calibrator are captured for image calibration. The projection matrix is then calculated through the image calibration. After the calibration, the 3D marked points are projected onto the 2D X-ray coordinate system using the projection matrix. The precision between the positioning arm and X-ray display is represented by comparing the position errors of the projected and actual image markers. In this study, we collected 15 experiments to calculate the accuracy of image calibration.

Accuracy of surgical positioning and arm positioning

To comprehensively assess our system's accuracy, we evaluated not only the error between the positioning arm and image but also the intrinsic positioning precision of the arm itself. This separate verification was conducted using a spine phantom embedded with steel ball bearings. The coordinates of each steel ball were meticulously established through a coordinate measuring machine, which served as the ground truth reference values. We equipped the positioning arm with a custom measurement attachment featuring a hemispherical groove specifically designed to mate precisely with the steel balls (Figure 6b). Using this setup, we measured the exact coordinates of steel balls by systematically touching each ball with the positioning arm's measurement attachment. The positioning precision of the arm was then determined by calculating the deviation between our arm-based measurements and the established ground truth coordinates. This approach allowed for direct quantification of the positioning arm's inherent accuracy. For statistical validity, we collected positional data from 15 steel balls per dataset and repeated this procedure to obtain three complete datasets.

Results

Positioning Accuracy

**Figure 9:**
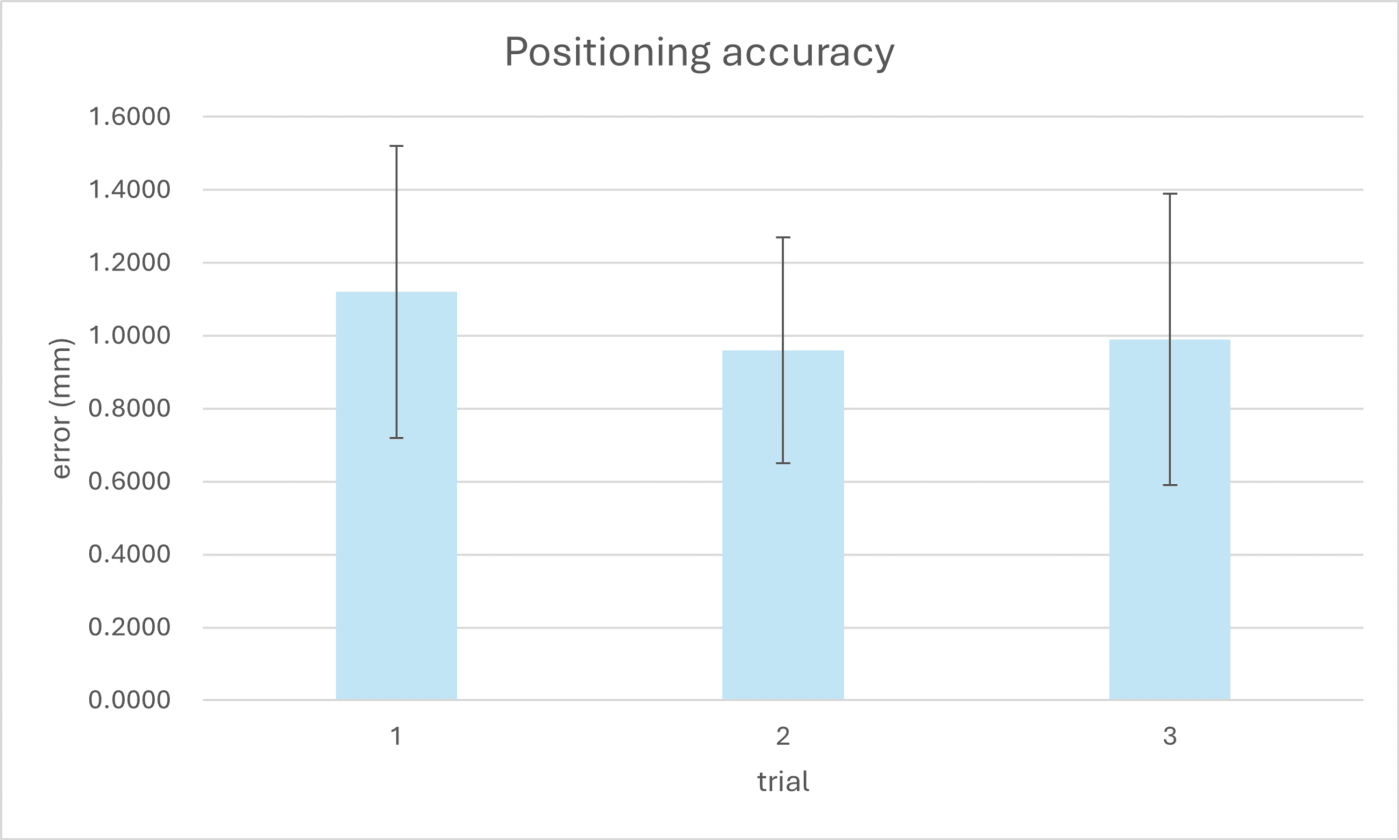
Positioning accuracy of the surgical arm across three independent experimental trials. Each bar represents the mean absolute positioning error (in mm) based on 15 steel ball measurements per case, with error bars indicating standard deviation. The results demonstrate consistent accuracy across repeated trials using the same phantom setup

The positioning accuracy of the arm was evaluated using three independent datasets, each comprising measurements of 15 steel balls embedded in a spine phantom. The 3D coordinates of each steel ball were first established using a high-precision measurement system (Inspector Classic 6.8.6, Hexagon AB, Sweden), which has a certified accuracy of ±0.01 mm, serving as the ground truth reference values. As shown in Figure 9, the mean absolute errors for the three experiments were 1.12 mm, 0.96 mm, and 0.99 mm, respectively, with standard deviations ranging from 0.31 mm to 0.40 mm. The first experiment exhibited the largest variability, while the second showed the most consistent results. Averaged across all datasets, the mean absolute positioning error was 1.03 ± 0.38 mm, confirming the system's capability to achieve low millimeter-level accuracy with acceptable repeatability.

**Figure 10:**
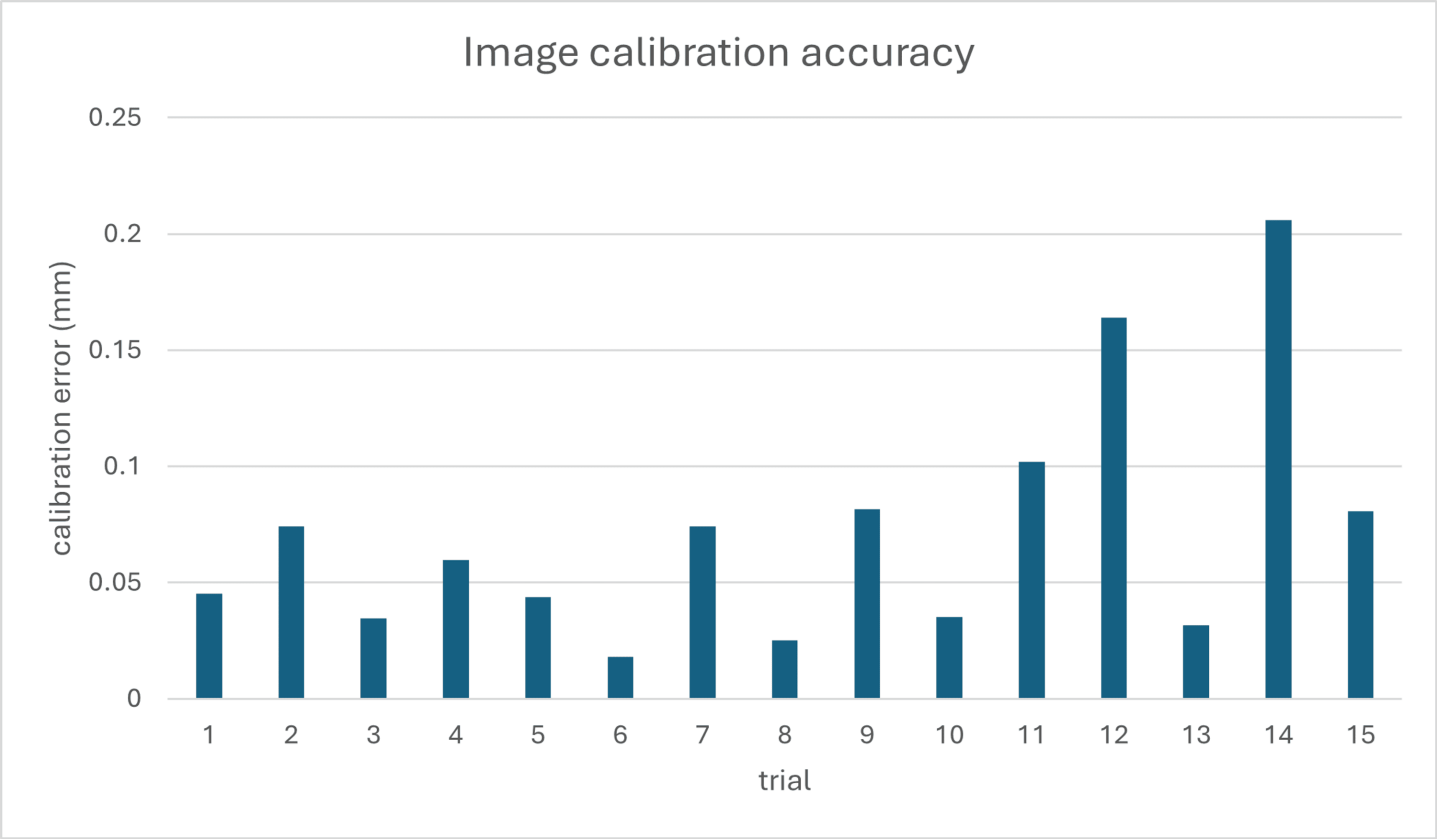
Calibration error analysis across 15 trials. The discrepancy between projected and actual 2D marker positions on the X-ray image is used to evaluate image calibration precision 2D: two dimensional

The image calibration accuracy was determined by calculating the projection matrix using markers on the customized calibrator. We compared the 2D marker coordinates transformed by the projection matrix with the actual marker coordinates on the X-ray image. As shown in Figure 10, a total of 15 independent calibration trials were performed. The calibration errors across experiments ranged from 0.02 mm to 0.20 mm, with most errors falling below 0.1 mm, indicating high consistency. The mean image calibration error across all trials was 0.07 ± 0.05 mm, demonstrating the system's high precision in image-space localization.

The combined positioning and calibration processes contribute to the overall accuracy of the system. By propagating the errors from both components, the overall device accuracy was determined to be 1.10 ± 0.43 mm. This represents the expected error when using the complete system for intraoperative guidance.

Surgical Time

The longer the duration of a surgical procedure, the higher the risk for the patient. Therefore, reducing surgical time is a critical issue. In this study, surgical time is divided into two components for evaluation: setup time and operation time. Setup time was defined as the period from the beginning of device positioning to completion of initial calibration, ready for surgical navigation. Operation time was defined as the duration from the start of the navigated intervention to its completion.

**Figure 11:**
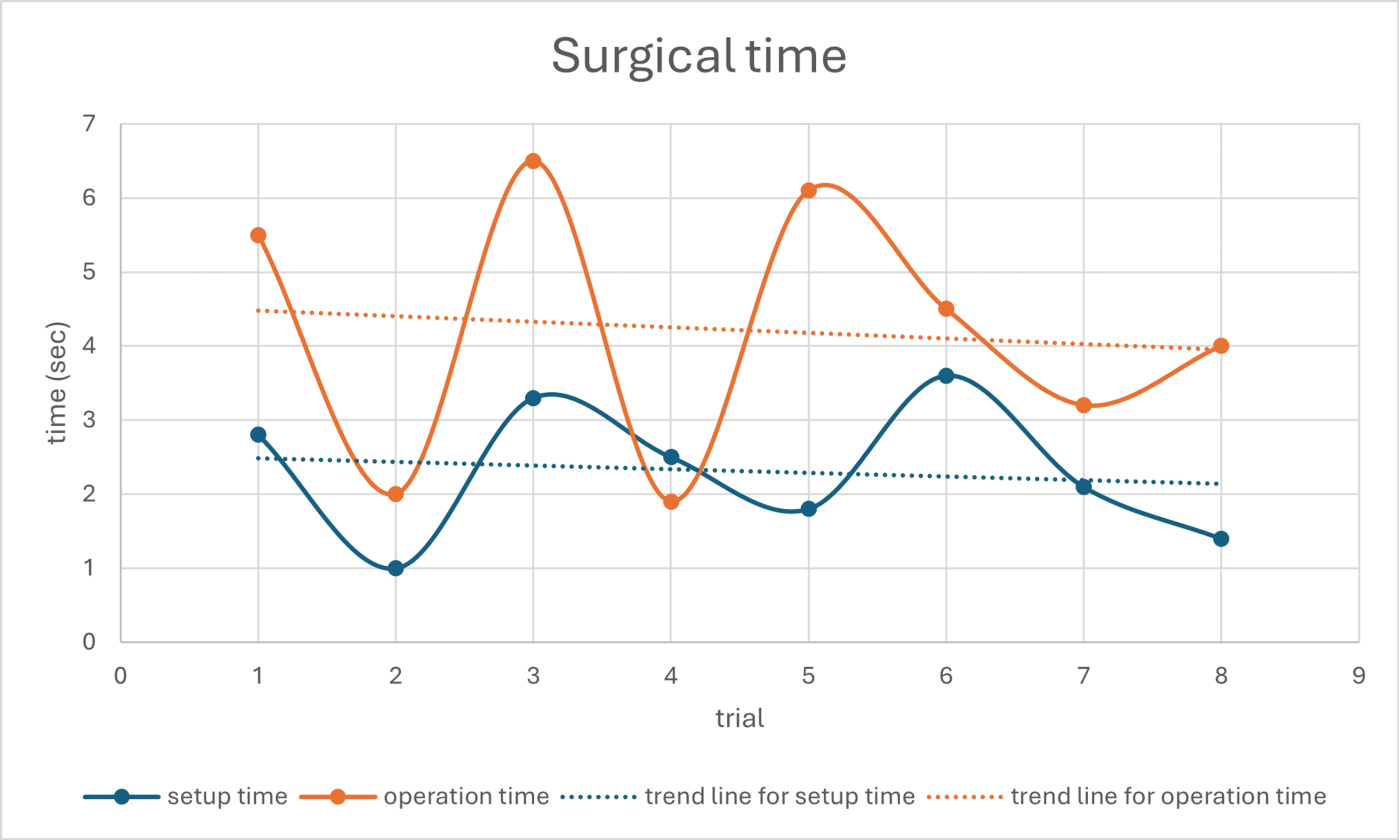
Setup and operation time recorded over eight procedures. Trend lines illustrate reduction in both durations over repeated trials, indicating increased familiarity and system efficiency

As shown in Figure 11, setup time and operation time were recorded across eight procedures. The setup time fluctuated between 1.0 and 3.6 minutes, while the operation time ranged from 1.9 to 6.1 minutes. Trend lines for both components are included to illustrate temporal patterns. Notably, both setup time and operation time exhibit a gradual downward trend, indicating improved efficiency and increased familiarity with the system over repeated trials. The average setup time across all tests was 2.3 ± 0.8 minutes, and the average operation time was 4.2 ± 1.6 minutes.

Radiation Exposure

In contemporary surgical practice, intraoperative radiation exposure has become a significant concern for both medical personnel and patients, as excessive radiation may lead to cumulative health risks and an increased likelihood of radiation-related diseases over time. Therefore, this study recorded the number and purpose of all radiographic images acquired during each procedure. The mean total number of C-arm images taken throughout the complete surgical process was 6.75±0.95 (range: 5-9). These images were categorized by purpose as follows: initial site identification, 4.25 ± 0.87 images (range: 4-6); system calibration, 1.50 ± 0.52 images (range: 1-2); and result confirmation, 2.00 ± 0.00 images (consistently two across all procedures).

Learning Curve and Usage Workflow

Our experimental observations indicate that the positioning system developed in this study provides a highly efficient and user-friendly workflow, with a minimal learning curve. Surgeons were able to seamlessly integrate the system into their surgical procedures without altering their usual practices. The positioning arm functioned as a supportive tool, enabling surgeons to maintain their grip on the instrument while receiving real-time positional feedback, which facilitated precise navigation during surgery.

The workflow for using the system was found to be straightforward. Once the equipment was calibrated using a simple X-ray image containing the customized calibrator, the same image could be utilized for continuous 2D navigation. This setup proved to be fast and effective, with no significant delays observed during the transition between calibration and navigation phases. Additionally, the compact size of the positioning arm contributed to its ease of use, making it more flexible and portable than other available surgical-assistance equipment.

These findings suggest that the system's ease of learning and operational efficiency can significantly enhance the overall surgical process without requiring extensive changes to existing practices.

## Discussion

Numerous previous studies have demonstrated that robot-assisted surgery can significantly enhance the safety and accuracy of surgical procedures [2,3,5,17]. According to the method we proposed, by mechanically positioning and utilizing sensors within seven joints to calculate the position of the end-effector and the target's 2D image, we can complete surgical navigation with real-time instrument projection on the screen, thereby addressing the challenges faced by existing navigation equipment. In the present study, we aimed to evaluate the effectiveness of our positioning system from five perspectives: system accuracy, surgical time, radiation exposure, learning curve, and usage workflow.

Positioning accuracy

The accurate placement of pedicle screws is crucial in spinal surgery, as high positioning accuracy leads to minimal screw deviation. Previous studies have reported various positioning accuracies for robotic and CT-guided systems. For instance, Chen et al. reported a positioning accuracy of 2 mm for their robot-assisted system [14]. In comparison, Jiang et al. found a mean screw tip deviation of 3.6 ± 2.3 mm in robot-assisted spine procedures [18]. Gubian et al. reported a positioning accuracy of 5.4 ± 2.6 mm for the CT-guided planning system [19]. In comparison, our system demonstrated a device accuracy of 1.10 ± 0.43 mm, which is superior to the performance of other robot-assisted and CT-navigated methods reported in the literature. This highlights the exceptional precision achieved by the proposed positioning system.

Surgical time

Time efficiency is a critical factor in evaluating the effectiveness of robot-assisted surgery and computer-assisted surgery. To provide a more precise evaluation of the time efficiency of the proposed system, we have separated the discussion into two components: setup time and operation time. In terms of setup time, Ding et al. reported a setup time of 28.3 ± 20.4 minutes before attachment of the reference frame. In contrast, Shin et al. reported an initial preparation time of 19 minutes for setting up the O-arm and navigation system [13,20]. In contrast, our system requires only 2.3 ± 0.8 minutes for setup, which includes mounting the positioning arm at the surgical bedside, connecting it to the workstation, and completing the system registration. Notably, there is no need for additional setup of optical devices or reference frame attachments, which significantly reduces the setup time compared to existing systems.

In terms of surgical time, Zhang et al. reported that the average time from anatomical registration to the insertion of the first pedicle screw using preoperative CT-guided navigation was 10.2 minutes [21]. Ding et al. reported that the average time required from reference frame attachment to completion of the first pedicle screw insertion was 22.3 ± 9.6 minutes [13]. In our study, the average operation time for completing the first pedicle screw insertion was 4.2 ± 1.6 minutes. Our system eliminates the need for instrument calibration, resulting in faster operation times compared to those reported by Zhang et al. and Ding et al.

Overall, our results demonstrate superior performance in both setup time and surgical duration with the new positioning system.

Radiation exposure

Reducing radiation exposure during surgery is crucial for protecting the health of surgeons and operating room staff, as prolonged exposure has been associated with a higher risk of cancer or DNA-damage-related illnesses [8,22]. In previous studies, it has been demonstrated that robot-assisted surgical systems and navigation systems can effectively reduce radiation time and decrease radiation exposure compared to traditional freehand surgeries [5,8]. To compare our result of radiation exposure with the calculated mean radiation exposure per pedicle screw, we divided the mean dose by the mean number of screws placed. Godzik et al. reported the mean cumulative radiation exposure per screw of 35.5 ± 28.6 mGy using fluoroscopy-guided surgery [23]. Dusad et al. reported that the mean cumulative radiation exposure per screw in the C-arm navigation and fluoroscopy-guided groups was 0.4 ± 0.1 mGy and 2.7 ± 0.6 mGy [24]. Khan et al. reported cumulative radiation exposure per screw as 10.4 ± 8.5 mGy for the O-arm navigation group and 15.8 ± 9.1 mGy for the O-arm robotic group [25]. Although this study did not directly measure the actual radiation exposure during surgeries using the positioning arm, we recorded the number of C-arm images taken throughout the surgical process. We estimated the radiation exposure by timing a 0.04 mGy radiation dose per C-arm image. Based on this estimation, the total radiation dose received during our surgical procedures, from positioning arm calibration to surgical completion, was approximately 0.27 ± 0.04 mGy, which had less radiation exposure than previous studies. Additionally, our positioning system eliminates the need for preoperative CT scans or intraoperative O-arm imaging, resulting in significantly reduced patient exposure to radiation compared to other navigation systems.

Learning curve

In terms of the learning curve, the method proposed in this paper does not change the surgeon's surgical habits. Still, it only provides auxiliary functions, similar to surgical support arms commonly used in surgeries. Surgeons only need to hold the instrument connected to the positioning arm to perform surgery and can instantly locate the instrument's position. Compared to optical navigation, which utilizes cameras to capture reflective markers, surgeons are limited by the camera's field of view and the occlusion of reflective markers. In contrast, the positioning arm method can address these issues and provide higher positioning precision. The navigation screen is also based on the traditional method of using an intraoperative C-arm to confirm surgical position, adopting a 2D image navigation approach that provides a continuous X-ray effect without requiring radiographic images, thus aligning closely with surgeons' existing surgical procedures.

Usage workflow

In terms of workflow, the images used for equipment calibration and navigation are shared. After completing equipment calibration through an X-ray image containing the customized calibrator, we can use the same image for 2D navigation. The overall setup is rapid and seamless, and with the small size of the positioning arm, our positioning system does not take up space, making it more flexible and portable compared to surgical-assistance equipment currently on the market (Figure 2a).

Future work

Despite the encouraging results of our current study, several methodological limitations should be noted. The use of sawbones models imposes inherent limitations when applying experimental results to clinical scenarios, requiring careful interpretation. Furthermore, our positioning system identifies opportunities for optimization, particularly in improving operational ergonomics.

Our subsequent research will systematically focus on enhancing the performance of the positioning arm, aiming to achieve significant improvements in both operational ergonomics and positioning precision. By adopting a comprehensive and meticulous approach, we plan to collect and rigorously analyze diverse clinical experimental data. The study's goal is to enhance its external validity and methodological rigor. These future investigations are essential for substantiating and expanding the theoretical and practical implications of our initial findings.

## Conclusions

In this study, we developed and evaluated a novel surgical positioning system designed to assist spinal procedures without relying on optical tracking. In controlled experiments using sawbone spine models, the system achieved a mean translational positioning error of 1.08 ± 0.40 mm, with an average setup time of 2.3 ± 0.9 minutes and a reduction in radiation exposure compared to conventional fluoroscopy-guided methods.

These results demonstrate the feasibility and potential advantages of the proposed system in improving surgical efficiency and reducing intraoperative radiation in a simulated environment. However, the study was limited to phantom-based testing and controlled conditions. Further validation, including cadaveric and clinical studies, is needed to assess its performance in real-world surgical settings and to refine the system for broader application.

Future work will focus on enhancing the system’s robustness and expanding evaluations to diverse surgical scenarios. Within the limitations of this preliminary study, our findings provide a foundation for developing more accessible, efficient, and lower-radiation alternatives to current surgical navigation technologies.
